# Progress in Medicine

**Published:** 1899-06-17

**Authors:** 


### Progress in Medicine.

### DISEASES OF THE RESPIRATORY SYSTEM.

(*Continued from page* 182.)

**Treatment of Asthma.**—Martin[4] says that in uncomplicated asthma sedatives will cut short the attack, such as the hypodermic injection of morphia (one-sixth to one-quarter grain), the administration of chloral, or the inhalation of chloroform. The effect of the last-mentioned is evanescent. Inhalation of nitre and other prepared powders is a popular remedy, but harm is often done by their indiscriminate use, as they predispose to the supervention of bronchial catarrh. Of remedies which may be continuously used, arsenic and iodide of potash are the most useful. Stramonium sometimes leads to paralysis of accommodation of the eyes, and some patients, especially those of a highly neurotic temperament, are peculiarly susceptible to it. Doses of even one-sixteenth grain of the extract will occasionally produce dryness of the throat, and a state of nervous excitement. Local conditions sometimes act as peripheral irritants in cases of asthma. The presence of a watery or mucoid discharge from the nose in cases of asthma other than hay asthma is not uncommon, but is not always associated with serious local disease. In such cases the insufflation of a solution of bicarbonate and chloride of sodium is often of much service, and the application of cocaine sometimes helps to relieve. Application of the cautery in some cases relieves hay asthma. Removal of large tonsils or adenoids in children does not of itself cure the asthmatic attacks. The removal of a nasal polypus may greatly lessen the attacks, but not a few cases persist after removal.

Diet needs regulating in many cases, especially where regular attacks occur after meals. Food should be very digestible, taken mostly in the earlier part of the day, and in moderate quantity. The patient should not, however, be starved, and indeed codliver oil often does great good. Goldschmidt[5] has much the same views on the treatment of an attack of asthma as Martin. Morphia is of great value, especially where the attacks are infrequent but pronounced. If morphia is not well borne chloral may be used. Amyl nitrate acts extremely well, and occasionally antipyrin and quinine may be useful. The attacks return after chloroform narcosis passes off. During the intervals iodides are of most service, but they are not merely useless but harmful in cases where expectoration is abundant.

**Emphysema.**—Gee[6] comes to the following conclusions as to the nature of emphysema. It is chiefly due to forced inspirations, although forced expirations may to some small extent play a part. The distension of the lung, in so far as it is due to inspiration, is secondary to distension of the chest. This forced inspiration is rendered necessary by a feeling of dyspnœa, which is consequent on obstruction of the air passages. In chronic progressive emphysema this obstruction depends on bronchitis, either humid and attended with free expectoration, or dry with scanty expectoration. When once emphysema is set up the dyspnœa and the necessity for forced inspiration are increased by the natural defect of expiratory power in the lungs and in certain parts of the chest, Nature having provided means insufficient for reducing excessive inspiratory distension to the normal, and for thus emptying the lungs of residual air. The degeneration and atrophy of lung tissue are usually dependent on preceding over-distension, although it is possible that pulmonary atrophy may to some extent be primary.

**Morbid Anatomy of Pulmonary Emphysema.**—Ginsburg[7] has investigated the histological changes in 30 cases of pulmonary emphysema. He concludes that the greatest change occurs in the elastic fibres, which are greatly hypertrophied, and present, also, a marked alteration in their reticular arrangement; this is most marked in the alveoli; the small bronchi, however, show the same arrangement although in a less pronounced degree. The change also shows itself in the shape and direction of the elastic fibres, for instead of being straight they assume a curved and bent form, so that they may easily be pulled asunder. Some of the fibres ultimately become distinctly granulated, probably owing to a fatty degeneration. There was no atrophy of the unstriped muscular fibres; they either remained quite normal, or were very slightly hypertrophied.

**Pneumonia.**—This, according to Symes,[8] is an acute specific fever; it is more than a pulmonary disorder. It resembles specific fevers in its mode of onset, in the course taken by the fever, and in the nature of the complications. Direct infection from one person to another is rare, but epidemics are not uncommon. The pyrexia of this fever may exist before there are any signs of lung implication, and such signs may never arise. Again, the pneumococcus invasion may spare the lung, and the disease manifest itself as a pleurisy, a tonsillitis, or as a middle ear suppuration. More commonly, together with pulmonary hepatisation, the commonest symptom of this infective process, there are other symptoms—pericarditis, otitis, meningitis, ulcerative meningitis, ulcerative colitis, or empyema. The suppurative lesions excited by the pneumococcus do not necessarily arise during the acute stage of pneumonia, but may occur when convalescence is well established. The best way of making certain of the presence of the pneumococcus is to draw up a little blood from the hepatised lung by means of an ordinary sterilised hypodermic needle thrust into the chest.

**Pneumonia in Infants.**—According to Northrup[9] the three best diagnostic signs of broncho-pneumonia in infants are the altered pulse respiration rate, the fever, and the presence of subcrepitant or crepitant râles in the chest. The respiration is not merely hurried, it is increased out of ratio to the heart rate; instead of 1 to 4 it might be 1 to 3, or even higher. The temperature is raised, either uniformly or intermittently. Dulness on percussion comes last as a diagnostic sign, and in small infants contributes little to early diagnosis.

**Foreign Bodies in Bronchi.**—Sevestre[10] shortly discusses the effect of foreign bodies in the bronchi. The physical signs in the earliest stage are those of obstruction to the entry of air into the lung; with a free entry there may be no signs present. Collapse of the lung below the obstruction soon ensues. If the bronchus which is plugged be large, the area of lung rendered functionless may be so great that a fatal result may ensue from the enormous amount of work thrown on the rest of the lung and on the heart; but this is a rare occurrence. If this does not occur, inflammation of the bronchi, with subsequent consolidation of the lung, takes place. The mucous membrane of the bronchus becomes ulcerated and bronchiectasis results; the consolidation of the lung around is a gradual process, and not usually the result of an acute pneumonia; it may go on to gangrene. In a few cases an abscess formed around the foreign body has worked its way to the surface and discharged, carrying the foreign body with it.

**Pulmonary Abscess.**—Godlee[11] says that the diagnosis of a pulmonary abscess is often most difficult. Theoretically it should give the signs of a cavity, but even if it communicate with a bronchus such an opening is often plugged. In such a case the diagnosis depends partly upon the history of the case and partly on the character of the expectoration. And even then it is often out of the question to say whether the cavity is in the lung or outside it. The surest method of localising the abscess is by exploratory puncture, which should be done by a bottle aspirator. A simple exploring trocar should not be used because, if pleural adhesions are absent, air may enter the pleural cavity in entering or withdrawing the needle, modify the physical signs, and, if the abscess has been reached, possibly infect the pleural cavity.

Valuable information may be obtained by examination of the expectoration. The material expelled from the mouth is never quite the same as that which leaves the cavity; it receives a variable amount of mucus from the air passages and saliva from the mouth, the amount depending chiefly on the amount of effort involved in coughing. If the expectoration is bile-stained it must have come from the liver, unless the patient is jaundiced. This suggests either hepatic abscess or hydatid of the liver. Fragments of hydatid cysts may occur, but do not help to localise the disease, especially in view of the fact that hydatids are often multiple. Small yellow bodies, looking like grains of iodoform, in the pus is characteristic of actinomycosis. Chocolate-coloured expectoration, consisting of pellets of pus, more or less darkly stained with blood, comes in nine out of ten cases from the liver, either abscess or hydatid. It should be examined whilst on the warm stage for the amœba coli. Dark brown and very offensive expectoration is met with in some cases of gangrene occurring in the course of acute pneumonia. As a rule, the pus expectorated is seen in the cup as more or less distinct nummular pellets, which either remain distinct or run together; the pus itself is non-aërated, but is mixed up with variable amount of more or less aërated mucus and saliva. The pus is often yellow or green, occasionally almost white. If it separates into two or three layers on standing, it suggests the existence of bronchiectasis. If the pus is occasionally streaked with blood the question of tubercle must be entertained. In cases of empyema hæmorrhage may occur on the abscess first bursting into the lung. The smell varies. In gangrene of the lung there is first a foul, earthy stink, which gradually becomes more offensive; in bronchiectasis the smell is foul and sickly. The chocolate pus from liver abscesses, and that from tuberculous abscesses, have seldom more than a faint, disagreeable smell. Cases of bronchiectasis offer a most unpromising field for surgery; in only one out of many operated on did a completely good result follow. The chronic non-tuberculous abscesses are the most favourable of all for operation. Gangrenous cases are very unfavourable, whilst localised pneumonic abscesses do very well.

In operating, the presence of an abscess having been ascertained by the aspirator, three inches of two or more ribs are first excised, then the lung is attached to the parietal pleura by six or eight stitches made in a ring by a curved Hagedorn's needle; an incision is made through the lung into the abscess, and a large tube is inserted which must be retained in position as long as there is any considerable quantity of discharge.

**Pyo-pneumothorax.**—Short[12] discusses the diagnosis between fluid and air above and below the diaphragm, *i.e.*, between supraphrenic and subphrenic pyo-pneumothorax. The history may be of assistance; in the supraphrenic variety the commonest cause is the giving way of a portion of pleura over diseased lung, and the disease generally is tuberculous deposit. Hence a previous history of cough, hæmoptysis, and night sweats will point to the supraphrenic variety. Again, an empyema opening into the lung, gangrene of the lung, pyæmic abscess of the lung, or wound of the chest, would each have a distinctive history. The subphrenic variety is commonly due to perforation of a gastric ulcer or secondary to a hepatic abscess. In the former case there may be a characteristic history of dyspepsia, though sometimes not. If the subphrenic abscess be on the right side suppurating hydatid or abscess of the liver must be especially thought of. Less common causes are perinephritic abscess and appendicitis.

If the onset is sudden the diagnosis lies between pneumothorax from early phthisis on the one hand, and a perforated gastric ulcer or hepatic ulcer on the other. In pneumothorax there is often a stabbing pain, coughing, and faintness, with great dyspnœa. In perforated gastric ulcer sudden acute pain is felt in the epigastrium, which remains acutely tender, and vomiting is commoner than in pneumothorax.

The main point to determine, in examining the patient, is the position of the diaphragm, which is not so easy, for it can only be judged from the position of neighbouring organs. In right-sided lesions, if the mischief be above the diaphragm, we should expect to get the liver pushed down and the heart driven over to the left. The dulness elicited in percussion over these organs would not be separated by any resonant area. If the mischief be subphrenic the liver may or may not be depressed, according to the amount of pus and air present. In left-sided lesions, if the mischief be above the diaphragm the heart is pushed to the right, and the normal area of cardiac dulness becomes resonant. Theoretically the spleen and left lobe of the liver should be pushed down, and occasionally they may be felt in a depressed position. In left-sided subphrenic cases the heart is raised, but is not pushed over to the right, unless fluid is also present above the diaphragm, a complication, however, which is not unusual. In all cases the presence or absence of movement in the diaphragm is of great importance. Mischief above the muscle usually depresses it, so that it fails to act. Mischief below may raise it, but at the same time increases the mechanical advantage at which it acts, and the movement continues, and can be recognised by its effect on the abdominal walls and their contents. Cases of subphrenic trouble are sometimes considerably complicated by fluid above the diaphragm as well. The commonest cause of subphrenic pyo-pneumothorax in the left side is perforation of a gastric ulcer.

**Pretuberculous Stage of Phthisis.**—Loomis[13] is of opinion that it is possible in many cases, especially in chloro-anæmics, to diagnose phthisis previous to the appearance of physical signs or of tubercle bacilli in the sputum. Weight, respiratory capacity, and chest measurement have no value in establishing the possibilities of the development of phthisis in themselves, but must be considered in relation to the height of the individual, when they furnish important aids to diagnosis. The comparative body-weight is obtained by dividing the weight (in pounds) by the height (in feet); in a normal man this should be 26, in a woman 23. The amount of air expired after a full inspiration is normally three cubic inches in a man, two cubic inches in a woman, for every inch of height. The mean measurement of the circumference of the chest, obtained by halving the sum of that at full inspiration and at full expiration, should never be less than half the height. Chloro-anæmia and persistent and unexplained disturbances of the digestive system are also symptoms of the pretuberculous stage of phthisis.

**Phthisis with Peculiar Physical Signs.**—Hale White[14] and Willmore,[15] a little time ago, reported two cases of phthisis in which the physical signs noticed during life were those of great displacement of the heart to the right; yet at both autopsies the heart was found to be normal and not displaced. Both observers accepted the inference that the displacement they diagnosed had been apparent only. Ewart[16] has recently suggested another explanation. These cases occur in fibroid excavating disease of the right lung. When at the same time the left lung and the heart are fixed by adhesions, the right side of the chest falls in. If the chest does not fall in, this must be due either to a considerable emphysematous distension of the right middle and lower lobes, or to the encroachment of the heart and left lung into the right side of the chest. Ewart, therefore, suggests that the explanation of these cases is, that when at the autopsy the sternum and costal cartilages were removed the heart dropped backward and to the left of its own weight, owing to the partial subsidence of the overstretched left lung; the discrepancy between the clinical and the post-mortem observations is apparent only. He suggests that in such a case skiagraphy might be employed during life, and the position of the heart ascertained after death by first examining it from the abdomen before air is let into the thorax.

[4] Martin, Brit. Med. Jour., Dec. 21, 1898. [5] Goldschmidt, Munich, 1898. [6] Gee, Brit. Med. Jour., Mar. 25, 1899. [7] Ginsburg, Vratch, No. 5, 1899. [8] Symes, Hospital, April 22, 1899. [9] Northrup, Med. Rec., Feb. 4, 1899. [10] Sevestre, Brit. Med. Jour., Jan. 21, 1899. [11] Godlee, Brit. Med. Jour., Jan. 21, 1899. [12] Short, Birmingham Med. Rev., 1899. [13] Loomis, Med. Rec., No. 24, 1898. [14] Hale White, Lancet, Nov. 19, 1898. [15] Willmore, Lancet, Dec. 10, 1898. [16] Ewart, Internat. Med. Mag., Mar., 1899.

